# A Rapid, Accurate and Machine-Agnostic Segmentation and Quantification Method for CT-Based COVID-19 Diagnosis

**DOI:** 10.1109/TMI.2020.3001810

**Published:** 2020-06-11

**Authors:** Longxi Zhou, Zhongxiao Li, Juexiao Zhou, Haoyang Li, Yupeng Chen, Yuxin Huang, Dexuan Xie, Lintao Zhao, Ming Fan, Shahrukh Hashmi, Faisal Abdelkareem, Riham Eiada, Xigang Xiao, Lihua Li, Zhaowen Qiu, Xin Gao

**Affiliations:** Computer, Electrical, and Mathematical Sciences and Engineering (CEMSE) Division, Computational Bioscience Research Center (CBRC)King Abdullah University of Science and Technology (KAUST) Thuwal 23955 Saudi Arabia; Department of BiologySouthern University of Science and Technology Shenzhen 518055 China; Cancer Systems Biology Center, China–Japan Union HospitalJilin University 12510 Changchun 130031 China; Peng Cheng Laboratory Shenzhen 518066 China; Heilongjiang Tuomeng Technology Company Ltd. Harbin 150040 China; Department of Computer TomographyThe First Affiliated Hospital of Harbin Medical University 74559 Harbin 150001 China; Department of Computer TomographyThe First Hospital of Harbin Medical University Harbin 150010 China; Institute of Biomedical Engineering and Instrumentation, Hangzhou Dianzi University 12626 Hangzhou 310018 China; Oncology CenterKing Faisal Specialist Hospital and Research Center 37852 Riyadh 11211 Saudi Arabia; Department Medical ImagingKing Faisal Specialist Hospital and Research Center 37852 Riyadh 11211 Saudi Arabia; Institute of Information and Computer Engineering, Northeast Forestry University 47820 Harbin 150040 China

**Keywords:** COVID-19, deep learning, segmentation, computerized tomography

## Abstract

COVID-19 has caused a global pandemic and become the most urgent threat to the entire world. Tremendous efforts and resources have been invested in developing diagnosis, prognosis and treatment strategies to combat the disease. Although nucleic acid detection has been mainly used as the gold standard to confirm this RNA virus-based disease, it has been shown that such a strategy has a high false negative rate, especially for patients in the early stage, and thus CT imaging has been applied as a major diagnostic modality in confirming positive COVID-19. Despite the various, urgent advances in developing artificial intelligence (AI)-based computer-aided systems for CT-based COVID-19 diagnosis, most of the existing methods can only perform classification, whereas the state-of-the-art segmentation method requires a high level of human intervention. In this paper, we propose a fully-automatic, rapid, accurate, and machine-agnostic method that can segment and quantify the infection regions on CT scans from different sources. Our method is founded upon two innovations: 1) the first CT scan simulator for COVID-19, by fitting the dynamic change of real patients’ data measured at different time points, which greatly alleviates the data scarcity issue; and 2) a novel deep learning algorithm to solve the large-scene-small-object problem, which decomposes the 3D segmentation problem into three 2D ones, and thus reduces the model complexity by an order of magnitude and, at the same time, significantly improves the segmentation accuracy. Comprehensive experimental results over multi-country, multi-hospital, and multi-machine datasets demonstrate the superior performance of our method over the existing ones and suggest its important application value in combating the disease.

## I. Introduction

COVID-19, the infectious disease caused by the severe acute respiratory syndrome coronavirus 2 (SARS-CoV-2), has become a global pandemic and the most urgent threat facing our entire species. It also posed a grand challenge to the scientific community to cope with the dire need for sensitive, accurate, rapid, affordable and simple diagnostic technologies.

SARS-CoV-2 is an RNA virus and belongs to a broad family of viruses known as coronaviruses. It consists of a positive-sense single-stranded RNA, and four main structural proteins, including the spike (S) proteins, the envelope (E) proteins, the membrane (M) proteins, and the nucleocapsid (N) proteins. Accordingly, there are two ways to detect the virus from patients’ samples: through the detection of the nucleic acids of the viru’ RNA or through the detection of the antibodies produced by the patients’ immune system. Therefore, in the latest guideline of Diagnosis and Treatment of Pneumonitis Caused by COVID-19 (the seventh version) published by the Chinese government, the diagnosis of COVID-19 must be confirmed by either the reverse transcription polymerase chain reaction (RT-PCR) or by gene sequencing.

However, due to the practical issues in sample collection and transportation, as well as the performance of the testing kits, especially at the initial presentation of the outbreak, such gold standards have been shown to have a high false negative rate. For example, among the 1014 COVID-19 patients in Wuhan up to February 6, 2020 [1], only 59% (601 out of 1014) had positive RT-PCR results, whereas 88% (888 out of 1014) had positive chest computerized tomography (CT) scans. Among the ones (601) with positive RT-PCR, CT scan also achieved a 97% sensitivity (580 out of 601). This suggests that CT scans can not only detect most of the positive ones by RT-PCR, but also detect a lot more cases (about 30% more in [1]).

Therefore, CT scans have been widely used in many countries and have particularly shown great success in China as one of the main diagnostic standards for COVID-19.

## II. Related Work

### A. Overview of CAD Systems for Lung Diseases

Imaging has long been used as the major diagnostic source for lung diseases, such as pneumonia, tuberculosis, and lung cancer. The most commonly used pneumonia imaging technologies are X-rays (or chest radiography) and CT scans [2]. X-rays provide flattened 2D images whereas CT scans provide cross-sectional images that can be used to reconstruct the 3D model of the lung.

With the advances in artificial intelligence (AI) and its applications in various fields, especially computer vision and imaging, AI has been widely applied to X-ray- and CT-based detection and diagnosis of pneumonia. AI-based computer-aided diagnosis (CAD) systems are shown to be able to provide fast detection and diagnosis, and, in some cases, perform equally or even more accurately than professional radiologists [2], [3]. A variety of methods have thus been developed in the past decade. From the input data point of view, the existing AI-based methods can be classified into three categories: the ones that take X-rays as inputs [4]–[8], the ones that take CT scans as inputs [9]–[14], and the ones that can handle both [15]. From the extracted feature point of view, some of the existing methods are based on manually crafted features [15], [16], whereas the majority of the remainders are based on automatically extracted features by deep learning methods [4]–[14]. From the machine learning model point of view, it is not surprising to see that most of the existing methods [4]–[9], [11]–[15] are based on convolutional neural networks (CNN) and its variants, which have achieved great success in computer vision and imaging tasks. In order to alleviate the insufficient data issue that commonly exists in biomedical imaging tasks, techniques like transfer learning [4], [6] and pre-training [15] have been applied.

### B. AI-Based CAD Systems for COVID-19

Although X-rays have been serving as the fastest and most easily accessible screening tool for diagnosing pneumonia, it has been shown that X-rays are inferior to CT scans in detecting COVID-19 patients because the indicative characteristics of COVID-19 pneumonia are only visible in 3D information, such as ground glass opacity (GGO) lesions in the peripheral and posterior lungs, and pulmonary nodules [1], [17], [18]. The fast reading speed and the high sensitivity of CT scans in detecting COVID-19 patients [1] make AI-based CAD systems based on CT scans an ideal approach to cope with the exponential expansion of the COVID-19 pandemic. A number of AI-based CAD systems have thus been very quickly developed and deployed as scientific efforts to combat this global crisis [18]–[23]. Due to the urgency of the needs, most of these methods are not focused on proposing novel, principled machine learning methods, but rather on quickly building a workable model by directly applying the off-the-shelf approach, e.g., CNN, ResNet, and inception networks.

Xu *et al.* [22] combined the CNN and ResNet models, and trained a screening system for COVID-19 on a CT scan dataset consisting of 110 COVID-19 patients, 224 Influenza-A patients, and 175 healthy people. Their model achieved a classification accuracy of 86.7%. In a similar study, Song *et al.* [21] applied a details relation extraction neural network (DRE-Net) model, called DeepPneumonia, and trained it on a CT image dataset with 88 COVID-19 patients, 101 bacteria pneumonia patients, and 86 healthy people, on which their model achieved an accuracy of 86% and AUC (area under ROC) of 0.95. Wang *et al.* [20] first tried to reduce the complexity of the problem by extracting region of interest (ROI) images from the CT scans, then extracted feature vectors by a modified inception network, and finally used fully connected layers to differentiate COVID-19 images from the typical viral pneumonia images. On a dataset with 1065 CT images with 30% being the COVID-19 images, their model achieved a classification accuracy of 89.5%.

Although identifying and classifying COVID-19 patients from CT scans are important and timely needed for diagnosis purposes, there is an impending need from the front-line clinicians to segment and quantify the infection volumes in patients’ lungs. Such information has been shown to be critical to not only the diagnosis, but also the prognosis and treatment of patients. For example, if a patient’s infection volume is higher than 50% of the entire lungs, the death rate is very high. On the contrary, if a patient’s infection only happens in one of the five lung lobes, the prognosis is very promising. However, among the various efforts on developing CAD systems for COVID-19 diagnosis, only a few of them can segment and quantify the infection regions from CT scans. Shan *et al.* [23] adopted a human-in-the-loop workflow, which starts from a small batch of manually segmented CT scans; then builds an initial model based on this batch and applies to the next batch; asks the radiologists to correct the segmentation; refines the model; and goes to the next iteration. Their machine learning model applies the 3D CNN that combines V-Net with the bottle-neck structure. Trained on 249 CT scans from 249 patients and tested on 300 CT scans from 300 patients, their active learning framework requires human experts to cut or add 9.17% of the final output to make the segmentation satisfactory.

Despite the great advances in developing AI-based CAD systems for CT-based COVID-19 classification, segmentation, and quantification, the existing methods, due to the urgent need for immediate clinical use, share one or more of the following bottlenecks. 1) They are trained and optimized towards certain datasets, which are often collected by the same CT machine, with the same parameters, and are annotated by the same radiologists. Thus, such models often become dataset-specific and lack generalization power on datasets from other machines, which hampers their practical usage. 2) The access to high-quality, annotated COVID-19 patients’ data are often limited and restricted, which cannot provide data-hungry deep learning models with sufficient training data, especially at the early stage of COVID-19 that needs the most urgent help from the AI systems. 3) Most existing methods can only conduct the classification of COVID-19 patients, but cannot provide the segmentation and quantification of the infection volumes, whereas the state-of-the-art method that is capable of doing so requires a high level of human intervention, which is difficult to satisfy, especially during the outbreak [23].

### C. 2.5D Methods for 3D Segmentation

Recently, there have been trends to use 2D methods to accelerate and improve the performance of 3D models on 3D segmentation tasks. In the literature, methods that fuse a stack of 2D segmentation models to get the 3D segmentation are called 2.5D models. Silver *et al.* [29] merged the segmentation results from nine different views and reached the state-of-the-art performance in 13 segmentation tasks over four different datasets. Zhou *et al.* [24] and Li *et al.* [25] both fused multiple 2D models to improve the training time and performance on 3D medical image segmentation tasks. They found that by taking the merits of 2D segmentation models, their 2.5D methods sometimes outperformed state-of-the-art 3D models like 3D U-Net. In general, 2.5D models have the following advantages: 1) simplicity for training and refinement: 2.5D models have much fewer hyper-parameters than the 3D models due to the much lower model complexity; 2) faster convergence rate: as 2.5D models usually have less parameters and lower memory requirement, they can often converge much faster than 3D models; and 3) faster prediction time: for example, [26] used 2D segmentation models to reduce the prediction time for 3D segmentation from 54 min to real-time.

### D. Contributions of Our Method

Segmenting the infection regions for CT scans of COVID-19 patients is a 3D segmentation problem with small data size, which motivated us to propose a 2.5D model for this problem. In this work, we propose a fully automatic, rapid, accurate, and machine-agnostic segmentation and quantification method for CT-based COVID-19 diagnosis. Our method has the following innovations: 1) to resolve the data scarcity issue, we propose the first CT scan simulator for COVID-19 by fitting the dynamic changes of real patients’ data measured at different time points; and 2) for this large-scene-small-object problem with limited data, we propose a novel algorithm to decompose the 3D segmentation problem into three 2D ones by using the symmetry properties of the lungs and other tissues, which reduces the number of model parameters by an order of magnitude and, at the same time, significantly improves the segmentation accuracy. Benefited from both innovations, our model performs very well on segmenting and quantifying infection regions from CT scans of patients, especially the early-stage ones, from multiple countries, multiple hospitals, and multiple machines, and thus provides critical information to the diagnosis, treatment, and prognosis of COVID-19 patients.

## III. Methods

### A. Overall Workflow

**Fig. 1.**
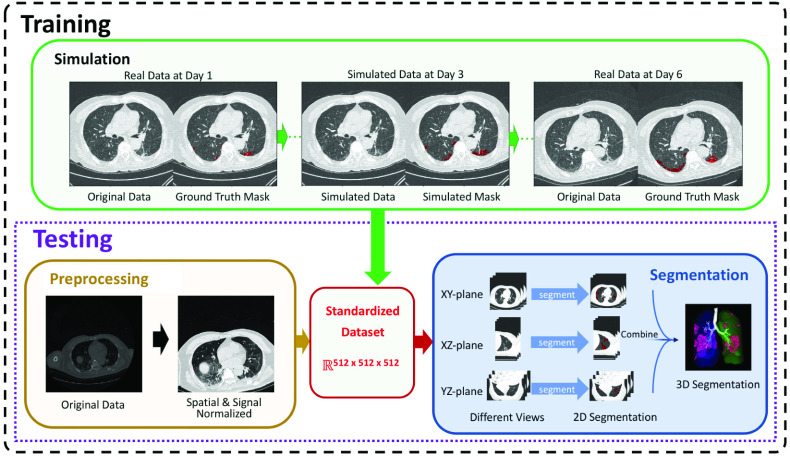
Flowchart of our CT-based COVID-19 segmentation and quantification method. First, we stack the CT scan images to a 3D tensor, and then normalize the resolution, signal intensities, and the dimension (i.e., casting to the standard embedding space). Our method then uses three 2D U-Nets to segment the infection regions along three orthogonal directions, and integrates the three segmented masks together to get the final infection segmentation. To train the data-hungry deep learning model, we further develop a data augmentation module to simulate the evolution of the infections, which can sample a large number of CT scans for the training purpose.

Fig. 1 illustrates the overall workflow of the proposed method. The task of infection segmentation is to find a mapping $\mathcal {F}:\mathbb { R}^{\mathrm {H\times W\times S}}\to {\{0,1\}}^{\mathrm {H\times W\times S}}$. Here $\mathrm {H\times W}$ is the image size of each CT image, and S is the number of images of the scan. Different CT scanners scan different volumes, and have different resolutions and parameters like $\mathrm {H,W}$ and S. Thus, we propose a data preprocessing method to embed any CT scan into a machine-agnostic standard space.

Deep learning models are data hungry while COVID-19 CT scan data are rarely available or accessible. Since our data contain multiple time-point CT scans of the same patient, we develop a dynamic model to simulate the progression of infection regions. Our simulation model can generate a large amount of training data, which is highly similar to the real data. The dynamic parameters of the simulation model are determined by fitting the model to the real data. The simulation model is then used to simulate 200 CT scans for each training sample, from which the augmented data are extracted. With the augmented data, our model achieves much higher performance.

The segmentation task is on 3D tensors each with ~^8^ voxels, and we only have limited training samples, even after data augmentation. Classical 3D segmentation models like 3D U-Net require a colossal number of training samples, and their prediction speed is too slow for clinical use, especially during this peak time of the COVID-19. To overcome this difficulty, we decompose the 3D segmentation problem into three 2D ones, along the x-y, y-z, and x-z planes, respectively. Our decomposition tactic achieves much higher performance than classical 3D segmentation methods and the state-of-the-art 2.5D models, and the prediction time is only several seconds per CT scan.

### B. Embedding to Standard Space

One of the main bottlenecks of the AI-based CAD systems is that they are trained on a certain dataset, and thus may not be directly generalizable to other datasets. In addition, when the input data come from different hospitals and are taken by different machines with different parameters, most existing methods cannot handle them directly.

**Fig. 2.**
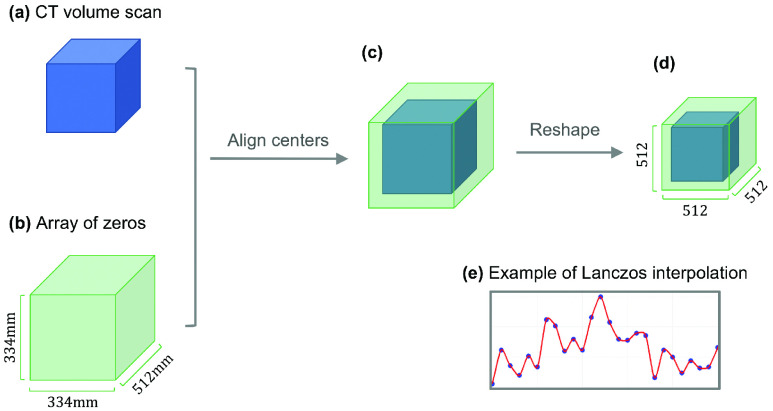
Illustration of the spatial normalization. (a) is the raw CT data while (b) has the same spatial resolution with (a), and (d) is the tensor after spatial normalization, which involves translation ((a)&(b) $\to $ (c)) and resizing ((c) $\to $ (d)). (e) Illustration of the reshape algorithm.

To overcome both issues, we propose a preprocessing method that can project any lung CT scan to the same, standard space, so that our model can take heterogeneous datasets as input, and can thus be machine-agnostic and applicable to any future dataset. Although preprocessing is a standard step in image analysis, to our knowledge, there is no method that simultaneously unifies the resolution, the dimension, and the signal intensity in CT image processing. Our preprocessing includes two normalization steps. The first one is the spatial normalization (Fig. 2), which unifies the resolution and the dimension of the CT scan; and the second one is the signal normalization, which standardizes the signal intensity of each voxel based on the lung windows of the CT scanners.

#### 1) Spatial Normalization:

the spatial normalization simultaneously unifies the resolution and the dimension. Different CT scans have different resolutions: for high-resolution scans, each voxel can correspond to a volume of $0.31\times 0.31\times 0.9 \mathrm {}{\mathrm {mm}}^{3}$, while for low-resolution scans, each voxel can represent $0.98\times 0.98\times 2.5{\mathrm {mm}}^{3}$. In our dataset, the norm of CT resolution is $\frac {334}{512}\times \frac {334}{512}\times 1.00{\mathrm {mm}}^{3}$, which is chosen as our standard resolution. We require that the standard embedding space $\mathcal {S}$ represents a volume of $334\times 334\times 512\mathrm { }{\mathrm {mm}}^{3}$, which is big enough to completely accommodate any human lung. Thus, $\mathcal {S}\in \mathrm { }\mathbb {R}^{\mathrm {512\times 512\times 512}}$.

Note that the invariant in our spatial normalization is the volume of each voxel (i.e., the standard resolution) in $\mathcal {S}$. During spatial normalization, the CT scan is first pad or cut into the volume of $334\times 334\times 512{\mathrm {mm}}^{3}$, and then use Lanczos interpolation [27] to rescale to the standard resolution (Fig. 2).

#### 2) Signal Normalization:

the values of CT data are in the Hounsfield Units (HU), which means that they are linearly normalized based on the X-ray attenuation coefficients of the water and the air. However, HU is suboptimal for lung CT scans, because the average CT values of lung parenchyma vary in different datasets (e.g., from −400 HU to −600 HU in our datasets).

In practice, experts set the lung window for each CT scanner and the types of human tissues in the lung window are approximately invariant for all scanners, e.g., the window level is around the average CT value of lung parenchyma. Two quantities, window level (WL) and window width (WW), are commonly used to describe this lung window. The WL is defined as the central signal value of this window, and the WW is the width of this window, which determines the difference between the upper bound value and the lower bound value.

Thus, we propose to use WL and WW to normalize the signal intensities: all voxels of $\mathcal {S}$ are undergone the linear transformation:

\begin{equation*} I_{normalized}=\frac {I_{original}\mathrm {-WL}}{\mathrm {WW}},\tag{1}\end{equation*}

where $I_{original}$ is the CT signal intensity of the raw data, and $I_{normalized}$ is the corresponding signal intensity after signal normalization. The signal normalization can be considered as an analog to the original Hounsfield normalization, which removes the machine-specific parameters for lung CT scans by setting the value of lung parenchyma to 0 and casting the values of human tissues in the lung window to the range of [−0.5, 0.5].

Thus, after spatial and signal normalization, any CT scan will be cast into a standard embedding space $\mathcal {S}$, which has the dimension of $\mathbb {R}^{\mathrm {512\times 512\times 512}}$, the resolution of $\frac {334}{512}\times \frac {334}{512}\times 1.00\mathrm {}{\mathrm {mm}}^{3}$ and the signal intensity range of [−0.5, 0.5].

### C. Data Augmentation

Deep learning models are data hungry, which request not only a large amount of but also high-quality annotated data for training. Unfortunately, in many applications, especially biomedical imaging, such data are rarely available or accessible. For example, the only publicly available image data collection for COVID-19 contains only X-ray data [28]. To overcome the lack of data bottleneck in deep learning applications, researchers have been using the idea of simulation, such as simulating Go games [29], simulating time-series fluorescence microscopy images [30], and simulating raw sequencing signals [31], [32].

**Fig. 3.**
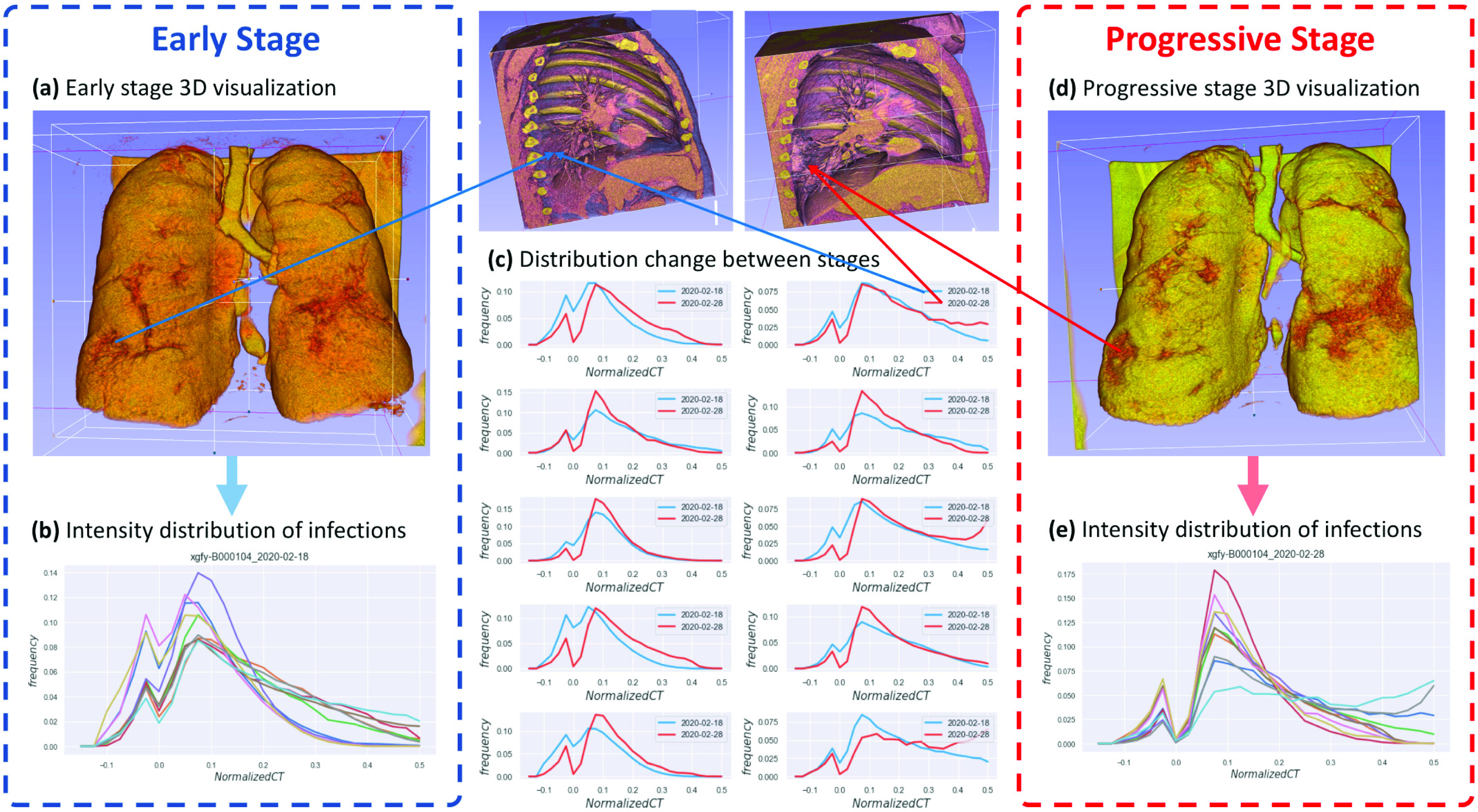
Illustration of the dynamic changes of the infection regions of a representative patient xgfy-B000104. (a) 3D visualization of the early stage lung. (b) The voxel intensity distributions of the 10 infection regions in (a). The x-axis is the voxel intensity value and the y-axis is the frequency. Each curve corresponds to one infection region. (c) The overlay of the curves from the early- and progressive-stages of the 10 infection regions, respectively. (d) 3D visualization of the progressive stage lung of the same patient. (e) The voxel intensity distributions of the 10 infection regions in (d). The x-axis is the voxel intensity value and the y-axis is the frequency. Each curve corresponds to one infection region.

The key to a successful simulator is to capture and to accurately quantify the underlying distribution that generates the real data. For our problem, although our main goal focuses on the diagnosis and segmentation of the CT scans from early stage COVID-19 patients, our dataset does contain multiple CT scans taken at different time points during a patient’s disease course, from which we can extract the statistics over time to build our simulation model. Fig. 3 illustrates the dynamic changes of the infections for a representative patient. The figure gives 3D visualization of how the infection progresses, and plots out the distribution of voxel intensities for infection regions.

We conduct the data augmentation through modeling and simulating the dynamic changes of the infection. The dynamic model has four basic components: how a new infection is generated; how an old infection is absorbed; how the normalized CT signals for infection voxels change; and how the normalized CT signals for normal voxels change. We first formulate our dynamic model, and then describe how to fit the parameters for the model and how to conduct the data augmentation.

#### 1) Model Formulation:

##### a) State:

the state of the dynamic model $\Psi $ is determined by the normalized data $\mathcal {S}\in \mathbb {R}^{\mathrm {512\times 512\times 512}}$, and the infection mask $\mathcal {M}\in {\{0,1\}}^{\mathrm {512\times 512\times 512}}$:

\begin{equation*} \Psi =\left ({\mathcal {S},\mathcal {M} }\right).\tag{2}\end{equation*}

##### b) Markov property:

considering that we only model a short period of time, state $\Psi $ should satisfy the Markov property. That is, we assume that within 24 hours of time, each infection region evolves for 100 times.

Denote the state at time point $t$ as $\Psi {}_{t}\mathrm =(\mathcal {S}_{t},\mathcal {M}_{t})$, and the transition function as $T$, thus $\Psi \Psi {}_{t+1}={\left ({\mathcal { S}_{t+1},\mathcal {M}_{t+1} }\right)=T(}_{t}\mathrm {)=(}{T_{\mathcal {S}}(\mathcal {S}}_{t}),{T_{\mathcal {M}}(\mathcal {M}}_{t}))$. We need to find a proper transition function $T$ so that during the evolution, the progression of the state (the augmented data) conforms to the real data, i.e., the CT scans for different time points of the same patient.

After extensive observations and analysis on our dataset, we have three interesting findings. First, for a CT scan, although there can be many disconnected infection regions, the distribution of the normalized voxel intensity for each infection region is highly conserved (Fig. 3b and Fig. 3e). Second, the frequency distributions for most infection regions have two clear peaks around −0.025 and 0. 1, and a clear valley around 0 (Fig. 3b and Fig. 3e). Third, when the infections deteriorate (e.g., from the early stage to the progressive stage, or from the progressive stage to the severe stage), the absolute number of voxels below 0 is much more stable than the rapid growth of the number of voxels above 0 (Fig. 3c).

With these findings, we further simplify the dynamic model. The first finding suggests that the dynamic model can be shared for all the infection regions. The second finding suggests that we should use different evolution functions to describe voxels with signals greater and less than 0. The third finding suggests that we can use simple functions like linear functions to describe the change of the absolute number of voxels below 0.

We thus determine $T$ according to these guidelines. We first determine $T_{\mathcal {M}}$, which depicts how new infection voxels are generated and how old infection voxels are absorbed. We have two assumptions about $T_{\mathcal {M}}$, which are discussed with and confirmed by the front-line radiologists. They are:

1) *Infection* model: normal voxels nearby GGO are more likely to become GGO voxels during the transitions of $\Psi $. In addition, normal voxels like areas outside the lung, tracheae, blood tubes, etc., will never become a part of infection regions.
2) *Recovery* model: when the signal intensity is smaller than −0.15, the voxel will become a normal voxel.

Next, we determine $T_{\mathcal {S}}$, which depicts how the signals change during the transition. We have four assumptions about $T_{\mathcal {S}}$, which are discussed with and confirmed by the front-line radiologists. They are:

1) Invariance for normal voxels: the signal intensities for normal voxels are invariant during the transition.
2) Absorption: the inflammatory infiltration is measured by the CT signal and our body absorbs the inflammatory infiltration at a constant speed.
3) Consolidation and fibration: when the CT signal increases, the voxel gradually consolidates, which means its signal becomes more difficult to further increase.
4) Threshold value 0: when the intensity of a GGO voxel reaches 0, its signal will not further increase. It has a probability to convert into the next stage and pass across value 0.

Our dynamic model has two major capacities. First, it can generate infection regions that comply with the signal intensity distribution of the real data for COVID-19. Second, it can generate infection regions that follow the morphology and spatial distribution of the real data, with user-controllable parameters. For example, we can require the GGO lesions to be more widespread, while the pulmonary consolidations to be relatively clustered.

The detailed algorithms for the transition function $T$ can be found in Section S1 in Supporting Materials. There are eight independent parameters for $T$. We denote the parameters of the dynamic model as $\mathbf {W}$, which is, $T=T(\mathbf {W})$.

#### 2) Fitting $W$ From Real Time-Series CT Scan Data:

We need two states: the starting state, $\Psi {}_{t_{i}}$, and the ending state, $\Psi {}_{t_{j}}$, to fit the dynamic parameters $\mathbf {W}$. $\Psi {}_{t_{i}}$ requires $t_{j}-t_{i}$ transitions to become $\Psi {}_{t_{j}}$. Thus, by applying the transition function $T(\mathbf {W})$ on $\Psi {}_{t_{i}}$ for $t_{j}-t_{i}$ times, we will get a simulated $\hat {\Psi }_{t_{j}}$. The difference between the signal intensity distribution of $\hat {\Psi }_{t_{j}}$ (denoted as $\hat {F}_{t_{j}}$) and that of $\Psi {}_{t_{j}}$ (denoted as $\mathrm {F}_{t_{j}}$) is used as the loss function to optimize $\mathbf {W}$:

\begin{equation*} L\left ({\mathbf {W} }\right)=\int {(\hat {F}_{t_{j}}-\mathrm {F}_{t_{j}})}^{2} \mathrm {dx.}\tag{3}\end{equation*}

Here $L\left ({\mathbf {W} }\right)$ is the loss function, and x is the CT signal. There are very fast algorithms to calculate $L\left ({\mathbf {W} }\right)$ without explicitly calculating $\hat {\Psi }_{t_{j}}$ which can be found in Section S1 in Supporting Materials. Fig. 3c gives examples of the change of the signal intensity distributions over time. By minimizing the loss, we can fit $\mathbf {W}$ for $\Psi {}_{t_{i}}$ and $\Psi {}_{t_{j}}$. More details of the fitting procedure can be found in Section S1 in Supporting Materials.

#### 3) Data Augmentation Through Simulation:

In our dataset, each patient has a scan from the early stage. We assume that three days before the earliest scan (denoted as time $t_{0}=0$), there is little infection. Denote the serial of scans and masks for a patient as:

\begin{equation*} {\Psi }_{t_{0}},{\Psi }_{t_{1}},\mathrm { }{\Psi }_{t_{2}},\ldots,{\Psi }_{t_{N}}.\tag{4}\end{equation*}

Here $\Psi {}_{t_{0}}$ is set as: $\mathcal {S}_{t_{0}}$ is gained by setting all infection voxels of the earliest scan $\mathcal {S}_{t_{1}}$ to −0.15 and $\mathcal {M}_{t_{0}}$ is gained by randomly selecting 10% of the infection voxels of $\mathcal {M}_{t_{1}}$. Since we define 100 transitions as per 24 hours of time and assume that the first CT scan happens 3 days after $\Psi {}_{t_{0}}$, we have $t_{1}=300$.

During data augmentation, we fit the parameter $\mathbf {W}_{i}$ for each pair of adjacent time points, $\Psi {}_{t_{i-1}}$ and $\Psi {}_{t_{i}}$, $1\le i\le N$, and apply $T(\mathbf {W}_{i})$ on $\Psi {}_{t_{i}}$ for 200 transitions to simulate CT scans for 200 time points. We then randomly select 1% of the simulated scans as the augmented data. Thus, the training samples are augmented by 200% through dynamic simulation.

It is worth noting that our data augmentation can be considered as an ‘interpolation’ method for CT scan time-series. However, instead of interpolating the morphologies of infection regions, it interpolates the infection volume and the signal distribution of infected voxels. We found that our method achieved the best performance at an augmentation ratio of 200% (Table V).

**TABLE I:** Imaging Protocols of 160 Patients From 2 Countries, 5 Hospitals, and 8 CT Scanners

	The Harbin Dataset						The Riyadh Dataset	
Hospital ID	A	A	B	B	C	D	E	E
CT scanner ID	1	2	3	4	5	6	7	8
Number of patients	50	10	13	7	55	5	17	3
Infection annotation	Voxel-level	Voxel-level	Voxel-level	Voxel-level	Voxel-level	Voxel-level	ROI-level	ROI-level
CT scanner	Brilliance iCT, Philips	Somatom Definition Edge, Siemens	Brilliance 16P CT, Philips	SOMATOM Scope, Siemens	GE 16-slice CT scanner	Brilliance iCT, Philips	Somatom Definition Edge, Siemens	Brilliance 16P CT, Philips
Number of Slices	256	64	16	16	16	256	128	16
Tube voltage (kVp)	120	120	120	110	120	120	100	140
Collimation (mm)	$128\times 0.625$	$128\times 0.6$	$16\times 1.5$	$16\times 1.2$	$16\times 1.25$	$128\times 0.625$	$128\times 0.6$	$128\times 0.625$
Matrix	$512\times 512$	$512\times 512$	$512\times 512$	$512\times 512$	$512\times 512$	$512\times 512$	$512\times 512$	$512\times 512$
Rotation time (second)	0.35	0.5	0.75	0.6	1.0	0.35	0.28	0.35
Pitch	1.0	1.2	0.938	1.5	1.75	1.0	1.0	1.0
Slice thickness (mm)	5	5	5	5	5	5	2	2
Slice increment (mm)	5	5	5	5	5	5	2	2
After thin-slice reconstruction								
Slice thickness (mm)	1	1	5	2	1.25	1	1	1
Lung window level (HU)	−600	−600	−600	−600	−550	−600	−400	−500
Lung window width (HU)	1600	1600	1200	1200	1500	1600	1400	1500

**TABLE II:** Scan Level Segmentation Performance

Methods	The Harbin dataset		
	dice	recall	worst-case
Ours	0.783 ± 0.080	0.776± 0.072	(0.577, 0)
2D U-net	0.565± 0.275	0.625± 0.292	(0.097, 13)
H-DUNet	0.597± 0.104	0.802 ±0.058	(0.124, 3)
MPUnet	0.449± 0.206	0.448± 0.190	(0.000, 31)
3D U-net	0.621± 0.112	0.702± 0.111	(0.032, 12)
3D V-net	0.641± 0.187	0.769± 0.123	(0.049, 8)
Ours*	0.783 ±0.080	0.776± 0.072	–
2D U-net*	0.593± 0.273	0.636± 0.291	–
H-DUNet*	0.605± 0.102	0.803 ±0.058	–
MPUnet*	0.559± 0.165	0.496± 0.155	–
3D U-net*	0.658± 0.105	0.707± 0.109	–
3D V-net*	0.667± 0.182	0.770± 0.123	–
Ours#	0.802 ±0.072	0.794± 0.068	(0.656, 0)
2D U-net#	0.617± 0.189	0.653± 0.202	(0.201, 0)
H-DUNet#	0.643± 0.095	0.823 ±0.042	(0.377, 0)
MPUnet#	0.543± 0.118	0.566± 0.095	(0.236, 0)
3D U-net#	0.706± 0.084	0.779± 0.75	(0.334, 0)
3D V-net#	0.708± 0.100	0.788± 0.71	(0.385, 0)
Ours@	0.903 ±0.037	0.898± 0.032	(0.728, 0)
2D U-net@	0.767± 0.169	0.787± 0.163	(0.224, 0)
H-DUnet@	0.820± 0.053	0.904 ±0.021	(0.477, 0)
MPUnet@	0.683± 0.138	0.660± 0.095	(0.254, 0)
3D U-net@	0.826± 0.084	0.849± 0.077	(0.424, 0)
3D V-net@	0.855± 0.055	0.887± 0.050	(0.503, 0)

The best performer under each criterion is in bold. The performance is shown in the form of average±standard deviation. The last column shows the worst-case dice and the number of failure cases (defined as dice below 0.2). The same 200% data augmentation was applied to train all the methods. Rows marked with * show the performance of the method by excluding the failure cases. Rows marked with # show the performance of the method by training and testing on the highest-quality subset from our dataset, which corresponds to the CT scans from the same machine with the highest signal-to-noise ratio, which were visually confirmed by radiologists. Rows marked with @ show the performance of the method by a less stringent evaluation criterion: as long as a predicted infection point is within 2 pixels from a true infection point, it is counted as a true positive. This criterion will make the prediction task much easier, especially for the early-stage patients.

**TABLE III:** Stage-Specific Segmentation Performance

Methods	The Harbin dataset		
	Early	Progressive	Severe
Percentage	73.1%	18.7%	8.2%
Ours	0.764	0.823	0.875
2D U-net	0.508	0.703	0.755
H-DUnet	0.526	0.788	0.803
MPUnet	0.370	0.618	0.766
3D U-net	0.551	0.802	0.820
3D V-net	0.581	0.796	0.824

The best performer under each criterion is in bold. The performance is shown in the form of average dice.

**TABLE IV:** Scan Level Quantification Performance

Methods	The Harbin dataset		
	RMSE	PCC	Worst-RSE
Ours	0.025	0.967	0.049
2D U-net	0.076	0.909	0.317
H-DUnet	0.072	0.927	0.304
MPUnet	0.092	0.825	0.276
3D U-net	0.056	0.948	0.167
3D V-net	0.048	0.943	0.175

The best performer under each criterion is in bold. The performance is shown in the form of average values.

**TABLE V:** Analysis of Data Augmentation for Different Methods

Methods	Augmentation Ratio					
	0%	50%	100%	200%	300%	400%
Ours	0.685	0.756	0.778	0.783	0.765	0.741
2D U-net	0.492	0.537	0.553	0.565	0.563	0.561
H-DUnet	0.526	0.557	0.581	0.597	0.590	0.585
MPUnet	0.433	0.442	0.453	0.449	0.457	0.445
3D U-net	0.571	0.606	0.630	0.621	0.609	0.597
3D V-net	0.593	0.622	0.649	0.641	0.611	0.595

The best performance for each method is in bold. The performance is shown in the form of average dice. All methods follow the same training/testing split. The augmentation ratio indicates for each CT scan in the training set, the ratio of the augmented data that was simulated. For example, 50% means that for each CT scan in the training set, one scan was simulated and was given 50% probability of being taken.

### D. Three-Way Segmentation Model

A CT scan is represented as a 3D tensor, for which the most intuitive idea would be to directly apply 3D deep learning models, such as 3D CNN and 3D U-Net. However, such 3D models are known to have various issues [25], including large numbers of parameters, slow convergence rates, and high requirements on memory. There have thus been efforts on decomposing the 3D segmentation problem into a series of 2D ones by taking slices along the z-axis (the direction of the body), but such strategy often has unsatisfactory performance due to the loss of information.

Here, we propose to decompose the 3D segmentation problem into three 2D ones, along the x-y, y-z, and x-z planes, respectively. Our idea is based on two facts. First, during our manual annotation along the x-y planes, when radiologists feel ambiguous about a voxel, they usually refer to images along the y-z and x-z planes to make the final decision. Thus, several 2D images from these three planes contain essential information about whether a voxel is an infection or not. Second, our normal tissues, such as lung lobes, pulmonary arteries, veins, and capillaries, have much more regular morphologies than infection regions. Their morphologies are more or less conserved among different patients, whereas patients’ infection regions can be completely different from each other. If a model only looks at one direction, say the cross-section x-y plane, then arteries or veins can be difficult to be differentiated from the infection regions, whereas if looking at the x-z or y-z planes, they can be easily differentiated.

Let us formulize the three-way segmentation model. Any lung CT scan is cast into $\mathcal {S}\in \mathbb {R}^{\mathrm {512\times 512\times 512}}$. For every voxel $\in \mathcal {S}$, there are three images: $\mathcal {P}_{\mathrm {xy}}^{\mathsf{s}}$ from the x-y plane, $\mathcal {P}_{\mathrm {yz}}^{\mathsf{s}}$ from the y-z plane, and $\mathcal {P}_{\mathrm {xz}}^{\mathsf{s}}$ from the x-z plane, so that $s=\mathcal {P}_{\mathrm {xy}}^{\mathsf{s}}\cap \mathcal {P}_{\mathrm {yz}}^{\mathsf{s}}\cap \mathcal {P}_{\mathrm {xz}}^{\mathsf{s}}$. Thus, the semantic of ${\mathsf{s}}$ can be considered as:

\begin{equation*} {p}^{\mathsf{s}}={\mathfrak {g}}\left ({\mathcal {P}_{\mathrm {xy}}^{\mathsf{s}},\mathcal {P}_{\mathrm {yz}}^{\mathsf{s}}\mathrm {, }\mathcal {P}_{\mathrm {xz}}^{\mathsf{s}}}\right),\tag{5}\end{equation*}

where $\mathrm {p}^{\mathsf{s}}$ is the probability that voxel ${\mathsf{s}}$ is an infection point. $\mathfrak {g}$ is the function to determine the voxel semantic from three orthogonal views. Directly training the model based on (5) is very time-consuming. Thus, we propose to use the approximation for (5):

\begin{align*} \hat {p}^{\mathsf{s}}=&g\left ({\hat {p}_{\mathrm {xy}}^{\mathsf{s}}\mathrm {, }\hat {p}_{\mathrm {yz}}^{\mathsf{s}}\mathrm {, }\hat {p}_{\mathrm {xz}}^{\mathsf{s}} }\right) \\=&g(f_{\mathrm {xy}}^{\mathsf{s}}(\mathcal {P}_{\mathrm {xy}}^{\mathsf{s}}),{f_{\mathrm {yz}}^{\mathsf{s}}(\mathcal {P}}_{\mathrm {yz}}^{\mathsf{s}}),f_{\mathrm {xz}}^{\mathsf{s}}(\mathcal {P}_{\mathrm {xz}}^{\mathsf{s}})).\tag{6}\end{align*}

(6) represents our three-way model architecture. Here $\hat {p}^{\mathsf{s}}$ is the predicted probability of ${\mathsf{s}}$ to be an infection voxel, and it is a real value; $f_{\mathrm {xy}}^{\mathsf{s}}$, $f_{\mathrm {yz}}^{\mathsf{s}}$, $f_{\mathrm {xz}}^{\mathsf{s}}$ are three intermediate models, and the inputs of these three models are information from x-y, y-z and x-z planes, respectively. Then the intermediate models output their predictions for the semantic of ${\mathsf{s}}$, and we denote their outputs as $\hat {p}_{\mathrm {xy}}^{\mathsf{s}}$, $\hat {p}_{\mathrm {yz}}^{\mathsf{s}}$, $\hat {p}_{\mathrm {xz}}^{\mathsf{s}}$, which are three real values. $g$ is the aggregation function for combining $\hat {p}_{\mathrm {xy}}^{\mathsf{s}}$, $\hat {p}_{\mathrm {yz}}^{\mathsf{s}}$, $\hat {p}_{\mathrm {xz}}^{\mathsf{s}}$ to get the final prediction $\hat {p}^{\mathsf{s}}$. The training of our model has two stages: the first one is to train intermediate models to calculate $\hat {p}_{\mathrm {xy}}^{\mathsf{s}}$, $\hat {p}_{\mathrm {yz}}^{\mathsf{s}}$, $\hat {p}_{\mathrm {xz}}^{\mathsf{s}}$ for every voxel ${\mathsf{s}}\in \mathcal {S}$; and the second one is to determine a reasonable $g$ for the final prediction.

#### 1) Intermediate Models:

Assume $\mathcal {P}_{\mathrm {xy}}\in \mathrm { }\mathbb {R}^{\mathrm {512\times 512}}$ is an image from an x-y plane of $S$, and assume a 2D segmentation model $f_{\mathrm {xy}}$ can segment infection pixels for any image from x-y planes. Thus, the output of $f_{\mathrm {xy}}(\mathcal {P}_{\mathrm {xy}})\in \mathrm { }\mathbb {R}^{\mathrm {512\times 512}}$ is the probability map of infections, which is the 2D array for $\hat {p}_{\mathrm {xy}}^{\mathsf{s}}$, ${\mathsf{s}}\in \mathcal {P}_{\mathrm {xy}}$. There are 512 different images along the x-y direction, so computing 512 times of $f_{\mathrm {xy}}$ will get $\hat {p}_{\mathrm {xy}}^{\mathsf{s}}$, ${\mathsf{s}}\in \mathcal {S}$. Similarly, we have a 2D segmentation model $f_{\mathrm {yz}}$ for images from the y-z direction, and $f_{\mathrm {xz}}$ for images from the x-z direction. By computing these three models, we get $\hat {p}_{\mathrm {xy}}^{\mathsf{s}}$, $\hat {p}_{\mathrm {yz}}^{\mathsf{s}}$, $\hat {p}_{\mathrm {xz}}^{\mathsf{s}}$ for every voxel ${\mathsf{s}}\in \mathcal {S}$.

We try many 2D segmentation architectures including U-Net, Mask R-CNN, etc. We also try to make $f_{\mathrm {xy}}$, $f_{\mathrm {yz}}$ and $f_{\mathrm {xz}}$ share some parameters. The experiments show that three independent U-nets have the fastest training time and perform the best. Thus, our intermediate models are three independent 2D U-nets.

After discussing with experienced front-line radiologists for COVID-19, we further improve our intermediate models. Although radiologists believe that by combining $\mathcal {P}_{\mathrm {xy}}^{\mathsf{s}}$, $\mathcal {P}_{\mathrm {yz}}^{\mathsf{s}}$ and $\mathcal {P}_{\mathrm {xz}}^{\mathsf{s}}$ they can determine whether a voxel ${\mathsf{s}}$ is infection or not, if we want to understand more detailed semantics like whether the infection is caused by H1N1 or COVID-19, they have to know more information from the adjacent images. In practice, they often check at least four extra images, which are the ones of −5, −2, +2 and +5 millimeters away from the voxel ${\mathsf{s}}$. Since the resolution of our standard embedding space is $\frac {334}{512}\mathrm {mm}$ for the x- and y-axes, and 1.0 0mm for the z-axis, images that are −5, −2, 0, +2, +5 millimeters away from the image containing the voxel ${\mathsf{s}}$ (denoted as the i-th image) are images i – 8, i – 3, i, i + 3, i + 8 along the x- or y-axis, and i – 5, i – 2, i, i + 2, i + 5 along the z-axis. We also try other combinations of this parameter and the performance is inferior to the combination of −5, −2, 0, +2, +5 (Table S1 in Supporting Materials). This idea is conceptually similar to dilated convolution, which aggregates information from the adjacent slices to effectively improve the performance [33].

Thus, based on experiments and clinical practice, the intermediate models $f_{\mathrm {xy}}$, $f_{\mathrm {yz}}$ and $f_{\mathrm {xz}}$ are three independent U-nets, which input five adjacent images (input dimension: $\mathbb {R}^{\mathrm {5\times 512\times 512}}$), and output the infection probability map for the central image (output dimension: $\mathbb {R}^{\mathrm {512\times 512}}$).

#### 2) Aggregation Function $\mathbf{g}$:

After the intermediate predictions $\hat {p}_{\mathrm {xy}}^{\mathsf{s}}$, $\hat {p}_{\mathrm {yz}}^{\mathsf{s}}$, $\hat {p}_{\mathrm {xz}}^{\mathsf{s}}$ for every voxel ${\mathsf{s}}\in \mathcal {S}$ are calculated, there are many ways to aggregate them together: linear combination with fixed or learnable weights, then taking a threshold; multiplying them together; using SVM with these three values as features, etc. After trying many choices, we find that the best performing $\mathbf {g}$ is a binary function, which simply sums up the intermediate predictions and then takes a threshold of 2: $\mathbf {g(}\hat {p}_{\mathrm {xy}}^{\mathsf{s}}$, $\hat {p}_{\mathrm {yz}}^{\mathsf{s}}$, $\hat {p}_{\mathrm {xz}}^{\mathsf{s}}\mathbf {)=(}\hat {p}_{\mathrm {xy}}^{\mathsf{s}}+\mathrm { }\hat {p}_{\mathrm {yz}}^{\mathsf{s}}+\mathrm { }\hat {p}_{\mathrm {xz}}^{\mathsf{s}}) > 2$ (Table S1 in Supporting Materials). This implies that normal tissues look different from infections in at least one plane.

### E. Performance Measures

To evaluate the segmentation performance, we use dice, recall, and the worst-case dice performance. Dice, or dice similarity coefficient (DSC), and recall are defined as:

\begin{align*} \mathrm {Dice}=&\frac {2\left \vert{ Y\cap Y^{\prime } }\right \vert }{\left \vert{ Y }\right \vert +\left \vert{ Y^{\prime } }\right \vert }, \tag{7}\\ \mathrm {Recall}=&\frac {\left \vert{ Y\cap Y^{\prime } }\right \vert }{\left \vert{ Y }\right \vert },\tag{8}\end{align*}

where $Y$ is the ground-truth infection region annotated by the radiologists, $Y'$ is the predicted infection region by a method, and $\vert Y\vert $ denotes the cardinality of the set $Y$. Both $Y$ and $Y'$ are binary tensors. It is known that for binary classifiers, the dice is the same as the F1-score. For COVID-19 diagnosis, recall is an important measurement because missing detection can cause fatal consequences of the patient and bring huge threat to the community. We further use the worst-case performance to indicate a method’s ability to generalize reliable prediction even in the worst-case scenario.

To evaluate the quantification performance, we use root mean square error (RMSE) and Pearson correlation coefficient (PCC), which are defined as:

\begin{align*} \mathrm {PCC}=&\frac {cov\left ({Z,Z^{\prime } }\right)}{\sigma _{Z}\sigma _{Z^{\prime }}},\tag{9}\\ \mathrm {RMSE}=&\sqrt {\frac {\sum \limits _{i=1}^{N} \left ({z_{i}-z_{i}^{\prime } }\right)^{2}}{N}},\tag{10}\end{align*}

where $N$ is the number of CT scans, $z_{i}$ is the ground-truth percentage of the infection volume to the lung volume of the $i$-th scan, $z_{i}^{\prime }$ is the predicted percentage of the infection volume to the lung volume of the $i$-th scan, $Z$ is the ground-truth percentage of all the scans, $Z^{\prime }$ is the predicted percentage of all the scans, $cov(Z,Z')$ is the covariance between $Z$ and $Z'$, and $\sigma _{Z}$ is the standard deviation of $Z$.

Finally, we compare the training and testing runtime and memory cost of different methods to assess their usefulness in meeting the needs of rapid diagnoses of COVID-19.

## IV. Results

### A. Data and Imaging Protocol

We collected 201 anonymized CT scans from 140 COVID-19 patients from 4 different hospitals, scanned by 6 different CT scanners, in Heilongjiang Province, China (hereinafter referred to as the *Harbin* dataset). In addition, to validate our method on a third-party dataset, we collected 20 anonymized CT scans from 20 COVID-19 patients, scanned by 2 different CT scanners, from King Faisal Specialist Hospital and Research Center (KFSHRC) in Riyadh, Saudi Arabia (hereinafter referred to as the *Riyadh* dataset). Since we are particularly focusing on early stage patients, we ensured that each patient has at least one CT scan from the early stage.

All the patients were confirmed by either the nucleic acid test or antibody test. The CT imaging protocols are shown in Table I. They represent a wide range of data varieties: the number of CT scans per patient ranges from 1 to 5; the age of the patients ranges from 19 to 87; the number of images per CT scan ranges from 245 to 408; the slice thickness after reconstruction ranges from 1mm to 5mm; the window width ranges from 1200HU to 1600HU; and the window level ranges from −600HU to −400HU.

The Institutional Biosafety and Bioethics Committees at KAUST, Harbin Medical University and KFSHRC approved this study, and the requirement for informed consent was waived due to the retrospective nature of this study.

### B. Imaging Segmentation

The lung region and the five lobes were automatically segmented by the Diagnostic Image Processing software developed by Heilongjiang Tuomeng Technology Co. Ltd. The infection regions were manually segmented by two radiologists with 20 years of experience, in consensus. The detailed segmentation protocol can be found in Section S3 in Supporting Materials.

The Harbin dataset was carefully segmented at a voxel-level. Since the infection areas often have higher density than the remaining parts of the lung, lung tissues with high density were manually checked and removed from the segmented infection areas, such as pulmonary arteries, pulmonary veins, and pulmonary capillaries. The Riyadh dataset was not segmented by radiologists at a pixel level, but rather at the region of interest (ROI)-level, denoted by circles. Therefore, the Harbin dataset was used for both quantitative and qualitative evaluation, whereas the Riyadh dataset was used for qualitative evaluation.

### C. Experimental Setup

For quantitative evaluation, we conducted 5-fold cross-validation (CV) over the Harbin dataset at the patient level, i.e., all the patients were randomly split into five folds, and each time, four folds were used for training and validation, and the remaining one was used for testing. If a patient was selected in a set, all of its CT scans were included in that set. All the compared methods were trained and tested on the same five-fold split to guarantee a fair comparison. To mimic the real-world application, the average scan-level performance was reported, instead of the patient-level one.

Since our dataset came from a variety of sources (Table I), we applied the same spatial and signal normalization before applying any compared method. After normalization, each scan was cast into the dimension of $\mathbb {R}^{\mathrm {512\times 512\times 512}}$ and the resolution of $\frac {334}{512}\times \frac {334}{512}\times 1.00\mathrm { }{\mathrm {mm}}^{3}$ for each voxel, and the signal intensity within the lung window was cast into the range of [−0.5, 0.5] according to Section III-B.

We applied data augmentation with different ratios over the Harbin dataset. That is, for each CT scan in the dataset, we simulated different numbers of scans as augmented data, according to Section III-C.

During the evaluation, we first fixed the augmentation ratio to 200% (i.e., for each CT scan, we simulated two scans) in Section IV-D & E, and trained all the compared methods on the same augmented datasets. We chose 200% for two reasons: 1) the majority of the compared methods obtained peak performance at this ratio (Table V), while the ones that did not (e.g., 3D U-net and 3D V-net) only had a small difference in performance between this ratio and the optimal one; 2) by fixing the augmentation ratio, we fairly evaluated the different segmentation models.

We then evaluated the detailed effects of data augmentation over different methods in Section IV-F. To this end, we augmented the data by 0%, 50%, 100%, 200%, 300% and 400%, where 50% means that for each CT scan, we simulated one scan and gave it 50% probability to be included in the training dataset. We thus obtained a comprehensive evaluation of the effect of our data augmentation strategy over different methods.

We compared our method with the baseline 2D segmentation method (i.e., 2D U-net over the x-y planes), the state-of-the-art 2.5D segmentation methods (i.e., MPUnet [34] and H-DenseUNet [25] (hereinafter referred to as H-DUnet)), the classical 3D method (i.e., 3D U-net [35]), as well as the backbone model of the available state-of-the-art segmentation method for COVID-19 (i.e., 3D V-net [23], [36]). Since the method in [23] is based on human-in-the-loop strategy, our implementation just tests its backbone 3D model, but cannot represent the actual performance of their method.

During the implementation of the 3D models, since the direct implementation consumes a huge amount of memory that none of our GPGPUs can accommodate, we divided the $512\times 512\times 512$ preprocessed CT scans into many sub-volumes shaped $128\times 128\times 128$ and fed each of them into the network independently. This is a common practice in 3D image processing, which does not affect the performance of 3D segmentation much, because most of the information for segmentation is well maintained in the sub-volume.

It is worth noting that for our method, we gave the users two outputs: 1) the binary prediction where 1 stands for infection and 0 stands for normal, and 2) the real-valued prediction which represents the probability of the voxel being infection. There are two reasons for this. First, through the discussion with the front-line radiologists, they felt that a tunable threshold to discretize such probability to binary prediction is practically useful for the clinical applications. Second, due to the high heterogeneity of our dataset, the huge number of possible morphologies of the infections, and the limited samples for COVID-19, the optimal threshold to convert the probability into the binary prediction over the training set may not be the same as the optimal one over the validation set (i.e., we split the four folds into training and validation for each iteration of the 5-fold CV). The same logic is applicable to all the compared methods as they can also output both real-valued (e.g., the output from the softmax layer) and discrete predictions. Therefore, we further tuned the threshold for all the compared methods over the same validation sets and selected the optimal threshold for each of them. All the evaluations were then done based on the discretized binary predictions after applying the corresponding thresholds.

### D. Segmentation Performance

We first set out to evaluate the segmentation performance of the proposed method. As shown in Table II, our method has a significantly higher dice than all the compared methods, improving the second-best method (3D V-net) by about 0.14, which demonstrates its superior performance on the voxel-level classification of the infection. Our method is able to identify most of the infection regions, demonstrated by a recall of 0.776, which is slightly lower than that of H-DUnet (0.802). However, H-DUnet achieved this recall at the cost of a large number of false positives. In addition, our method is not only accurate, but also robust: the worst-case performance in terms of dice is 0.557, whereas H-DUnet failed on 3 cases (dice below 0.2) and other methods failed on even more cases. MPUnet seems quite unstable and failed on many cases, which conforms to their reported performance and high variance on large-scene-small-object tasks such as tumor segmentations (e.g., Tasks 1, 3, 6, and 10 in Table I in [34]).

The results in Table II suggest that our 2.5D model significantly outperforms other 2.5D models (i.e. MPUnet and H-DUnet), which seems to be counter-intuitive as our three-way model is conceptually simpler than the compared 2.5D models. There are two main reasons for this. 1) The number of parameters of other 2.5D models is more than five times higher than that of our model (Table VI second column). The majority of applications of 2.5D models in image segmentation focus on the small-scene-large-object scenario. However, the CT scan segmentation for COVID-19, especially for early-stage scans, is a typical large-scene-small-object problem with limited data, thus models with an overwhelming amount of parameters cannot learn effectively. 2) Our data contain CT scans from different machines with different protocols. In fact, 2D U-net, H-DUnet, MPUnet, 3D U-net and 3D V-net failed in segmenting the infection regions on 13, 3, 31, 12, and 8 cases, respectively, which badly influenced their overall performance. A detailed inspection reveals that these failed cases are mostly scans with artifacts or have tiny infection regions. If such cases are not counted, existing methods can achieve much better performance (Table II second block).

**TABLE VI:** Runtime and Memory Consumption

Methods	# Parameters	Prediction	Training	Memory
Ours	8.6M	15 sec	5.5 h	128 GB
2D U-net	2.9M	10 sec	2 h	64 GB
H-DUnet	49M	~18 min	20 h	128 GB
MPUnet	62M	~12 min	24 h	128 GB
3D U-net	4.1M	~3 min	10 h	128 GB
3D V-net	12M	~3 min	9 h	128 GB

The best performer under each criterion is in bold. The four columns are: number of parameters for the deep learning model (M: million); prediction time for one patient; training time on the Harbin dataset; and the memory demand during training.

To further validate this, we repeated the experiments on the highest-quality and less-variant subset of the Harbin dataset which was collected from the same CT machine of the same hospital (i.e., CT scanner ID ‘1’ from hospital ID ‘A’ in Table I). The subset contains CT scans of 50 patients taken by a 256-slice Brilliance iCT, Philips, and has the highest signal-to-noise ratio in our dataset, which was visually confirmed by radiologists. We conducted 5-fold cross-validation (Table II third block). Comparing to the performance over the entire dataset (Table II first block), the performance of our method is stable and robust, whereas the other methods have clear improvement in terms of both dice and recall.

The reported performance of the segmentation methods in Table II might seem to be inconsistent with some recent studies, such as [23]. There are three possible reasons for this. First, our dataset contains a mixture of different stage scans, the majority of which are early-stage ones (73%). In general, the early-stage segmentation is much more challenging than the progressive- and the severe-stage segmentation because of the scattered and small infection regions, no clear boundaries for many infection regions, and the high variance in the infection volumes (e.g., the infection region volume of one early-stage scan can be more than 500 times bigger than that of another early-stage scan). Second, the ground-truth of our dataset is based on very detailed manual segmentation that excludes tracheae and blood-vessels inside infections, which makes voxel-level dice a highly stringent evaluation metric. To validate this, we used a less stringent evaluation criterion. That is, as long as a predicted infection point is within 2 pixels from a true infection point, it is counted as a true positive. This criterion will make the prediction task much easier, especially for the early-stage patients. Using this criterion for evaluation, the average dice of the existing methods improved by at least 0.2, whereas that of our method improved by only about 0.12 (Table II fourth block vs. first block). This suggests that our method is capable of predicting scattered and tiny infection regions, which is critical to segment infections from the early-stage patients. Third, a very recent publication [37] reported the average dice for different segmentation models to be around 0.55, which is consistent with our reported values and demonstrates that the absolute dice values highly depend on the datasets, and thus the relative comparison among different methods is more important.

We then conducted a more detailed analysis on different methods’ performance over the early-, progressive- and severe-stages. As shown in Table III, the existing methods performed reasonably well on the progressive- and severe-stages. On the most difficult stage, the early-stage, our method outperformed the existing methods by a larger margin, i.e. more than 0.18 increase in dice comparing to the second-best method, 3D V-net. This illustrates the power of our method in segmenting early-stage patients.

**Fig. 4.**
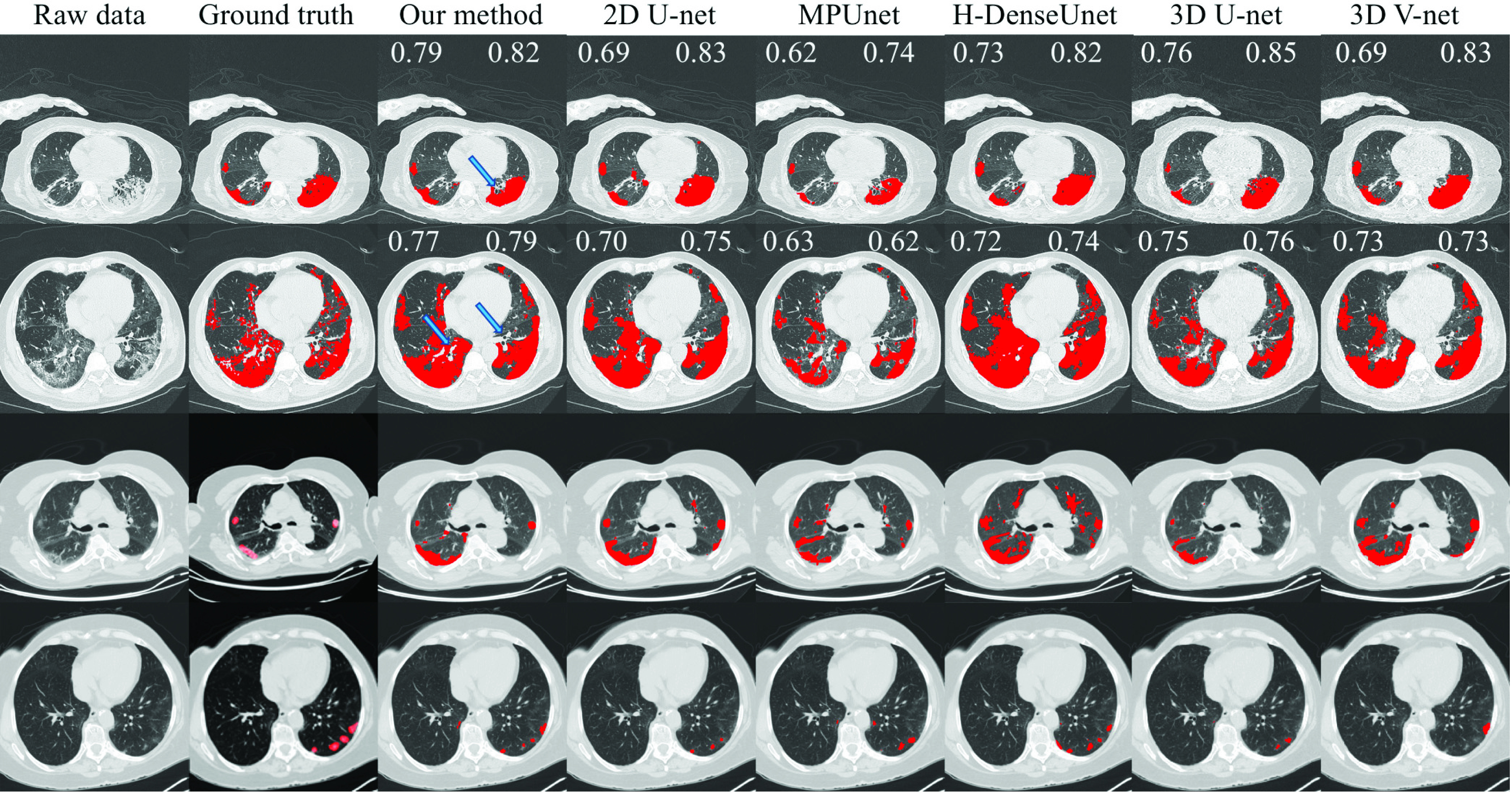
Illustration of the performance of different methods on representative patients from the Harbin dataset (the first two rows) and the Riyadh dataset (the last two rows). The eight columns represent the raw data, the ground-truth segmentation, the segmentation results by our method, by 2D U-net, by H-DUnet, by MPUnet, by 3D U-net, and by 3D V-net, respectively. For the two examples on the Harbin dataset, since we have voxel-level annotations of the ground-truth, we reported the dice values for both the scan-level (top left corner) and the single-image-level (top right corner) for each method. The blue arrow in the first example indicates a trachea that was mistakenly marked as infection by the manual annotation. The two blue arrows in the second example indicate arteries and tracheae.

Fig. 4 shows four representative examples of the segmentation results for different methods from the Harbin dataset and the Riyadh dataset. It can be seen that our method consistently performed well on these examples, whereas the compared methods sometimes under-segmented and sometimes over-segmented the infection regions. For the second example, our method can correctly segment the majority of the large infection regions while distinguishing arteries and tracheae embedded in the regions (indicated by blue arrows). Interestingly, for the first example, our method also distinguished one possible trachea (indicated by the blue arrow) in the infection region, whereas the manual annotations considered that as the infection. After consulting experienced radiologists, that region is indeed a trachea.

### E. Quantification Performance

We then evaluated the quantification performance of different methods by comparing the RMSE and Pearson correlation coefficient between the actual percentage of the infection volume to the lung volume, and the percentage of the predicted infection volume to the lung volume. This percentage has been shown to provide critical information for the treatment and prognosis of COVID-19 patients.

Table IV shows that our method provides highly accurate quantification to the infection volume, with an average error rate of only 2.5%, which is much lower than the second best method. The worst-case error rate of our method is 4.9%, whereas the worst-case error rate of other methods is at least 16% and can be as high as 31%. This significant outperformance is due to the accurate segmentation of our model and its ability to correctly distinguish lung tissues such as arteries and veins from infection regions.

### F. Augmentation Analysis

To comprehensively evaluate the effect of data augmentation, we applied different augmentation ratios on the training data and reported the performance of all the compared methods in Table V. It is clear that all the 2D, 2.5D and 3D methods can significantly benefit from data augmentation, which suggests the potential of our data augmentation approach being a general strategy to boost the state-of-the-art segmentation methods for COVID-19.

We observed that different methods achieved the peak performance at different augmentation ratios. In general, the 2D and 2.5D methods tend to benefit more from a higher augmentation ratio (e.g., 200%) than the 3D methods (e.g., 100%), although the difference for ratios above 100% seems to be small. This makes sense because the 2D and 2.5D models take less information as inputs than the 3D models, thus it is highly challenging for them to distinguish lung lobes, pulmonary arteries, veins, capillaries and artifacts. Data augmentation can greatly help and reinforce them in correctly eliminating such false positive predictions. On the other hand, our data augmentation approach does not create information, but rather interpolates the infection volumes and distributions, while estimating the morphologies for new infections. Thus an overly high augmentation ratio will not further boost the performance.

### G. Runtime and Resources Comparison

We further compared the time and memory consumptions of different methods. As shown in Table VI, our method cost less than 6 hours to train on 4 GPU cards of GeForce GTX 1080, which is much lower than the other 2.5D methods and 3D methods. A similar conclusion can be drawn in terms of the prediction time. The prediction time of our method is even comparable to that of the 2D method, which, again, confirms that our segmentation model provides a good tradeoff between time and accuracy. All together, these results demonstrate the efficacy of our segmentation model, i.e., decomposing the 3D segmentation problem into three 2D ones.

### H. Case Studies

**Fig. 5.**
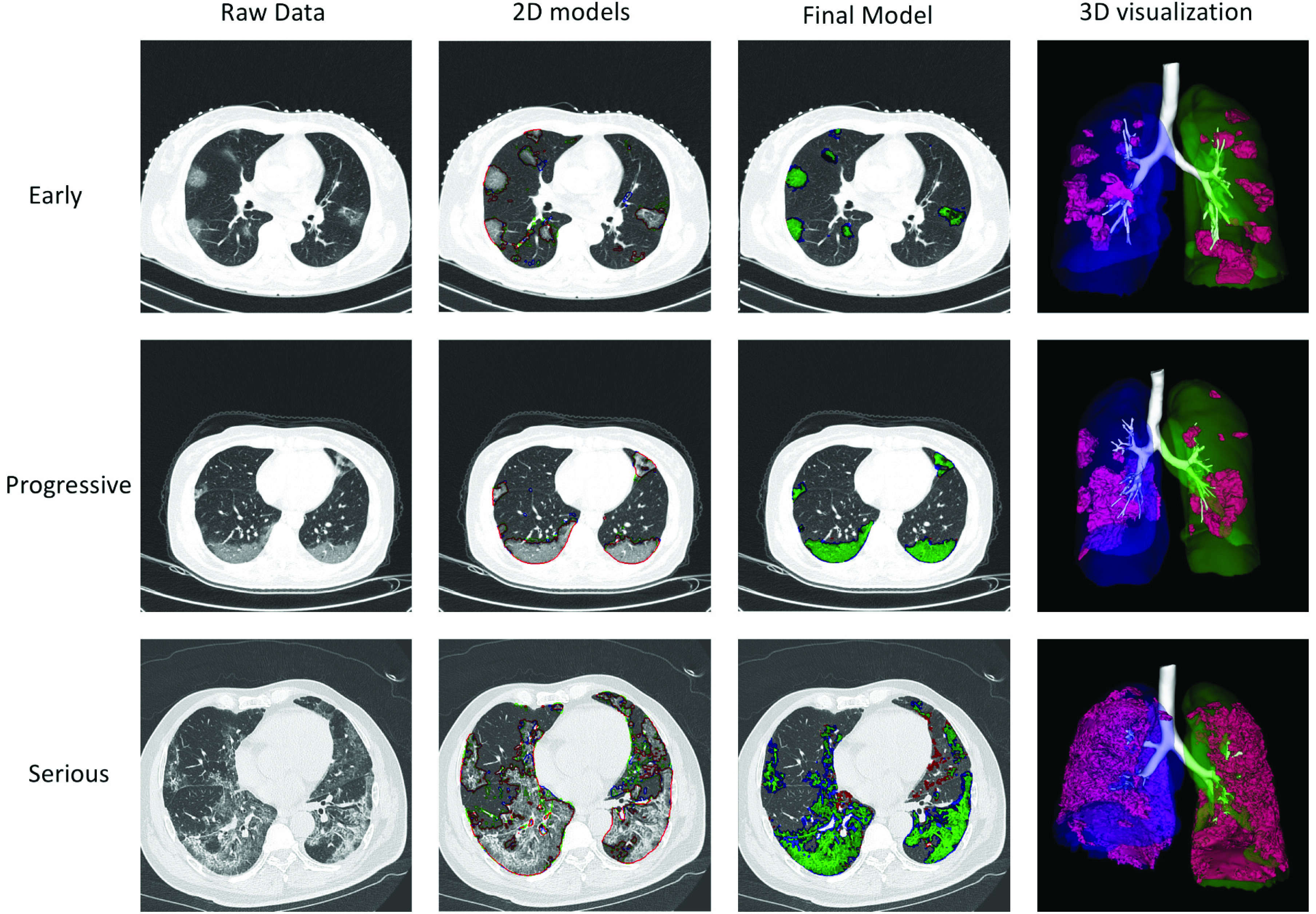
Case study of three representative patients from the Harbin dataset, one at the early stage, one at the progressive stage, and one at the severe stage. The first column shows a representative image of the raw CT scan. The second column shows the segmentation results of the three 2D models for this image: red stands for the 2D model for the x-y plane, green stands for the 2D model for the y-z plane, and blue stands for the 2D model for the x-z plane. The third column shows the segmentation results of our final model, where green stands for true positive, red stands for false negative, and blue stands for false positive. The fourth column shows the 3D visualization of the segmentation results of our model.

Three representative cases for the early-, progressive- and severe-stages are shown in Fig. 5. It can be seen that the three 2D segmentation models each cannot achieve an accurate segmentation result. However, they provide complimentary information to each other. Thus, their combination enables our model to not only segment the infection regions, but also correctly remove the tracheae and blood vessels from the infection.

### I. Ablation Studies

We conducted ablation study to investigate the contribution of the three components to the success of our method. As shown in Table VII, removing any component decreased the dice significantly. Among the three components, the integration of multiple 2D models is the most important one, followed by data augmentation.

**TABLE VII:** Ablation Study on the Harbin Dataset

Our method	-Normalize	-Combine	-Augment
0.783	0.712	0.67, 0.64, 0.63	0.685

The best performer is in bold. The four columns are: the dice using all three components; the dice without normalization to the standard space (simply padding to the standard dimension); the dice of using only 2D model from one view (the three values show the x-y view, the x-z view and the y-z view, respectively); and the dice without data augmentation

**Fig. 6.**
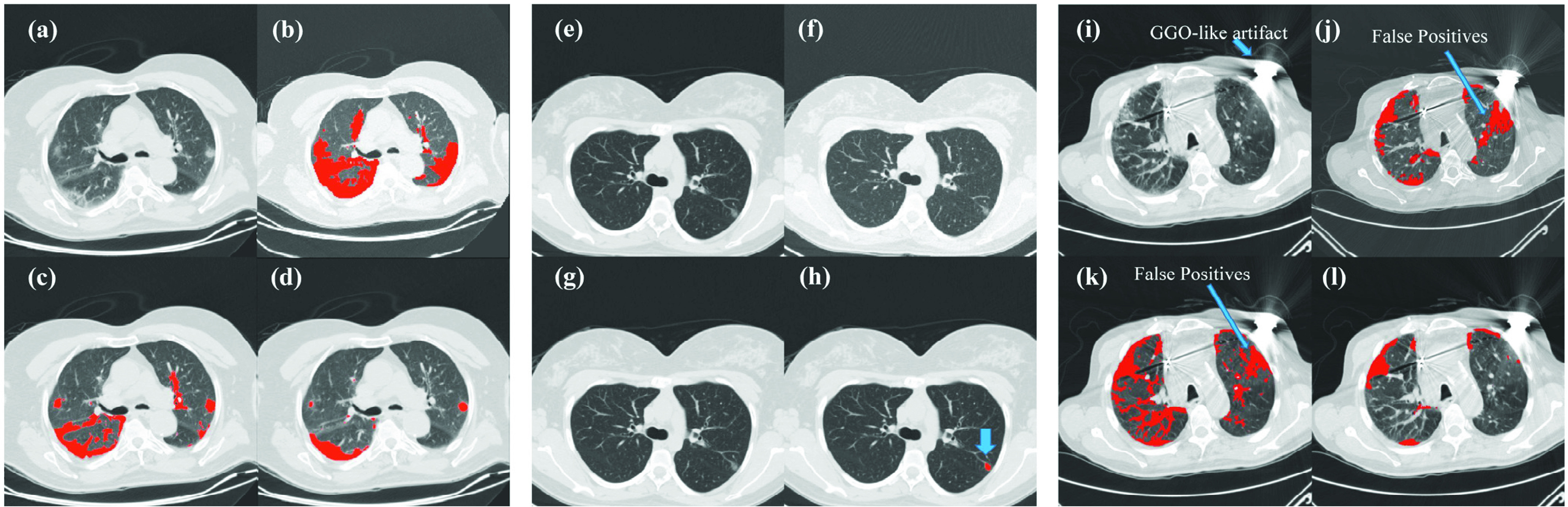
Ablation study of three representative patients from the Riyadh dataset, where (a)- (d) represent the first one, (e)- (h) represents the second, and (i)- (l) represent the third. (a)&(e)&(i): raw images of the CT scan; (b)& (f)& (j): segmentation results without preprocessing; (c)& (g)& (k): segmentation results without data augmentation; (d)& (h)& (l): segmentation results with all three components of our method. The third example contains a large number of artifacts in the raw image, which is possibly due to the metal implants in the patient during the CT scanning. The blue arrows in (h): our predicted infection region, in (i): GGO-like artifacts, and in (j) and (k): false positive predictions.

Three representative patients from the Riyadh dataset were used to further demonstrate the contribution of the different components of our model. The model trained on the Harbin dataset was directly applied to the Riyadh dataset, without re-training. As shown in Fig. 6, without the preprocessing step, the model becomes very unstable for data generated from other machines, sometimes generates a large number of false positives (Fig. 6b and Fig. 6j) and sometimes fails to segment anything (Fig. 6f). Data augmentation also contributes to the success of our method. Without it, the model can falsely segment blood veins (Fig. 6c and Fig. 6k) or fail to find any infection region (Fig. 6g). Combining all components together, our model was able to provide a consistently accurate segmentation (Fig. 6d, Fig. 6h and Fig. 6l). Among the three examples, the third one contains a large number of artifacts (Fig. 6i), possibly due to the metal implants in the patient during the CT scanning. Both the preprocessing and data augmentation components helped our model greatly in removing false positive predictions.

## V. Conclusion

In this work, we proposed a preprocessing method to cast any lung CT scan into a machine-agnostic standard embedding space. We developed a highly accurate segmentation model on the standard embedding space. To train the model, we further designed a novel simulation model to depict the dynamic change of infection regions for COVID-19, and used this dynamic model to augment extra data, which improved the performance of our segmentation model.

The preprocessing method resolves the heterogeneity issue in the data and makes our method applicable to any dataset generated by any CT machine. The segmentation model finds a good tradeoff between the complexity of the deep learning model and the accuracy of the model. In addition, it indirectly captures and incorporates the regular morphologies of lung tissues, such as lung lobes, pulmonary arteries, veins, and capillaries. This makes our model both accurate and rapid. Interestingly, we noticed that our model can sometimes outperform human annotations when distinguishing tracheae and blood vessels. We used a similar segmentation idea for a recent project on segmenting breast tumors from DCE-MRI images. The two studies thus suggested that this idea could be a generic approach for many biomedical imaging tasks, which requires further investigation and confirmation. The simulation model resolves the commonly-seen data scarcity issue for biomedical imaging tasks, particularly for COVID-19, where high-quality, annotated data are rarely accessible or available. These three cornerstones contribute together to the success of our method.

The comprehensive experiments on multi-country, multi-hospital, and multi-machine datasets showed that our segmentation model has much higher dice, recall, and worst-case performance, and runs much faster than the state-of-the-art methods. Our model thus provides a fully-automatic, accurate, rapid, and machine-agnostic tool to meet the urgent clinical needs to combat COVID-19.

There are three main directions to further improve our method. The first is to develop a federated learning platform. During our data collection process, we noticed that many hospitals have COVID-19 patients’ data but due to various reasons, they are not allowed to share the data with outside researchers. Thus, federated learning is an ideal solution to this situation, where we can train the model across different hospitals while each of them holds their own data and no data exchange is required. The second one is to further increase the size of the dataset. Despite the efforts in collecting heterogeneous data and developing preprocessing approach, our current dataset size is still limited. More data will bring more information and thus lead to better models, which is our ongoing work. The third one is to incorporate orthogonal sources of information to the model, such as big epidemiology data, so that ambiguous cases can be better diagnosed, and the source and spread of the cases can be better traced. When the outbreak ends, such a multimodal learning platform can be used as a long-term warning system to serve as a ‘whistleblower’ to the future coronavirus yet to come.

The program of our method is publicly available at: https://github.com/lzx325/COVID-19-repo.git. The CT scan datasets are available upon request.
